# A Comparative Study on Physiochemical, Thermomechanical, and Electrochemical Properties of Sulfonated Poly(Ether Ether Ketone) Block Copolymer Membranes with and without Fe_3_O_4_ Nanoparticles

**DOI:** 10.3390/polym11030536

**Published:** 2019-03-21

**Authors:** Ae Rhan Kim, Dong Jin Yoo

**Affiliations:** 1 Department of Bioenvironmental Chemistry and R&D Center for CANUTECH, Business Incubation Center, Chonbuk National University, Jeollabuk–do 54896, Korea; canutech@hanmail.net; 2 Graduate School, Department of Energy Storage/Conversion Engineering and Hydrogen and Fuel Cell Research Center, Chonbuk National University, Jeollabuk-do 54896, Korea; 3 Department of Life Science, Chonbuk National University, Jeollabuk–do 54896, Korea

**Keywords:** porous structure, SPEEK, hydrophobic oligomer, Fe_3_O_4_, tensile strength, proton conductivity

## Abstract

The composite structure, good porosity, and electrochemical behavior of proton exchange membranes (PEMs) are important characteristics, which can improve the performance of polymer electrolyte fuel cells (PEFCs). In this study, we designed and synthesized an XY block copolymer via a polycondensation reaction that contains sulfonated poly(ether ether ketone) (SPEEK) (X) as a hydrophilic unit and a fluorinated oligomer (Y) as a hydrophobic unit. The prepared XY block copolymer is composed of Fe_3_O_4_ nanoparticles to create composite architecture, which was subsequently treated with a 1 M H_2_SO_4_ solution at 70 °C for 1 h to eliminate Fe_3_O_4_ and generate a pores structure in the membrane. The morphological, physiochemical, thermomechanical, and electrochemical properties of bare XY, XY/Fe_3_O_4_-9 and XY(porous)-9 membranes were measured and compared in detail. Compared with XY/Fe_3_O_4_-9 composite, the proton conductivity of XY(porous)-9 membrane was remarkably enhanced as a result of the existence of pores as nano-conducting channels. Similarly, the XY(porous)-9 membrane exhibited enhanced water retention and ion exchange capacity among the prepared membranes. However, the PEFC power density of XY(porous)-9 membrane was still lower than that of XY/Fe_3_O_4_-9 membrane at 60 °C and 60% relative humidity. Also, the durability of XY(porous)-9 membrane is found to be lower compared with pristine XY and XY/Fe_3_O_4_-9 membranes as a result of the hydrogen crossover through the pores of the membrane.

## 1. Introduction

Environmental pollution is a severe threat to all countries owing to increasing emanation of green-house gases from the incineration of fossil fuels [1,2,3]. On the other hand, fuel cells are a green energy technology that converts chemical energy directly into electrical energy in a single step [4]. Among various types of fuel cells, polymer electrolyte fuel cells (PEFCs) are being considered as a promising technology because of their high power density, high conversion efficiency, and rapid start-up and shut-down times [5,6]. Proton exchange membranes (PEMs) are gaining popularity in PEFCs because of their favorable proton conductivity and gas barrier properties. For example, Nafion is the state-of-the-art membrane used in PEFCs because of its long-term durability, high electrical insulation, high proton conductivity, excellent dimensional stability, and good chemical stability. Regardless of its benefits, the proton conductivity of Nafion decreases dramatically at high temperatures or low relative humidity (RH) because of its inability to retain water molecules [7].

Recently, researchers have developed various alternative PEMs consisting of aromatic hydrocarbon polymers, such as sulfonated poly(imide), sulfonated poly(arylene ether sulfone) sulfonated poly(phenylene), and sulfonated poly(ether ether ketone) (SPEEK) [8]. Although the hydrocarbon polymer-based PEMs exhibit admirable performance, their usage come with a difficult trade-off between ion conductivity and dimensional stability [9]. Exploiting block copolymer PEMs consisting of both hydrophilic and hydrophobic units is a fascinating strategy that has been proposed to alleviate the trade-off between ion conductivity and dimensional stability [10]. In general, the synthesis of a block copolymer involves nucleophilic substitution between oligomers and the subsequent polymerization process, which results in the formation of block copolymer structure with extended polymer chains [11]. In a block structure, the ionic channels are generated as a result of phase separation, which can facilitate the transport of protons. Additionally, inorganic–organic composite PEMs have gained the attention of researchers because of their advantages in mechanical strength and thermal stability [12]; the composite PEMs can combine the advantages of both the organic matrix and the inorganic fillers. Various types of inorganic fillers, including SiO_2_, TiO_2_, ZrO_2_, Fe_3_O_4_, CeO_2_, and Al_2_O_3_, have been exploited as additives to enhance the comprehensive performance of PEMs [13]. Specifically, Fe_3_O_4_ nanoparticles have the advantages of high electrical conductivity and electrocatalytic activity, thus it has been widely used in various electrochemical applications. Also, the –OH groups on the surface of Fe_3_O_4_ make good interactions with polymer chains that improve the membrane’s properties [14,15,16]. On the other hand, the creation of porous structures in aromatic membranes is one of the best strategies to enhance the water uptake in membranes [4]. Materials that contain ordered and interconnected nano-pores have efficient and selective proton transport, which is a critical benefit in PEFCs. State-of-the-art Nafion is a well-known porous membrane that possesses connective proton transport channels [17]. As they comprise the optimal arrangement of proton transport sites, connective proton transport channels are distributed, which results in high proton conductivity.

Inspired by the previously mentioned properties, in the present work, we created pores in the membranes by incorporating and subsequently dissolving 30 nm Fe_3_O_4_ nanoparticles. We report the synthesis of an XY block structure, wherein X was the SPEEK as a hydrophilic unit and Y was the fluorinated poly(arylene isopropylidenediphenol biphenyl) oligomer as a hydrophobic unit using nucleophilic substitution strategy. Further, the composite membrane was fabricated by blending the XY block copolymer with 30 nm Fe_3_O_4_ nanoparticles. The porous membrane was then created by dissolving Fe_3_O_4_ nanoparticles by immersing composite membrane into 1 M H_2_SO_4_ at 70 °C for 1 h. The successful formation of SPEEK, hydrophobic oligomer, and XY block copolymer were verified with proton nuclear magnetic resonance (^1^H NMR) analysis. The effects of pores on structural, morphological, thermal, and mechanical properties of membranes were also examined using corresponding analytical techniques. The physiochemical properties were evaluated in terms of water uptake, swelling ratio, ion exchange capacity, and water contact angle to show the improvements due to the porous structure of the membrane. The electrochemical properties were investigated in terms of proton conductivity and PEFC performance to test the suitability of the porous membrane for use in fuel cell applications. We have compared all the obtained physiochemical, thermomechanical, and electrochemical properties of membranes with and without Fe_3_O_4_ to find better PEMs for PEFC application.

## 2. Experimental Section

### 2.1. Materials

Poly(ether ether ketone) (PEEK) powder was purchased from Victrex Company, South Korea. 4,4′-hexafluoroisopropylidenediphenol (HFIP) and decafluorobiphenyl (DFBP), N,N-dimethylacetamide (DMAc), N,N-dimethylformamide (DMF) dimethyl sulfoxide (DMSO), N-methylpyrrolidinone (NMP), anhydrous potassium carbonate (K_2_CO_3_), and toluene were derived from Sigma-Aldrich, South Korea and used without further purification. Other reagents and solvents were exploited as obtained.

### 2.2. SPEEK (X) and Hydrophobic Oligomer(Y) Synthesis

To produce the X component, we utilized the same reaction conditions and work-up procedures as those given in the literature [18]. The prepared X component was then immersed into a 0.1 M NaCl solution to exchange H^+^ with Na^+^. The Na salt form of X was dried and stored for later use. Then, 2.017 g (6.0 mmol) of HFIP, 2.0 g (6.0 mmol) of DFBP, 1.66 g (12.0 mmol) of K_2_CO_3_, 11 mL of DMAc, and 20 mL of toluene were charged together in a three-necked round-bottomed flask that was fitted with all of the required equipment. The reaction was performed at 120–160 °C in an oil bath under a N_2_ atmosphere with constant stirring (300 rpm). The solid product was cleaned with a mixture of methanol, acetone, and deionized (DI) water to remove the low molecular weight substances. Subsequently, the obtained component Y was dried and stored for later use.

### 2.3. XY Block Copolymer Synthesis

A total of 0.714 g (5.0 × 10^−3^ mmol) of the Na salt form of X was mixed and magnetically stirred under a N_2_ atmosphere with 0.123 g (5.0 × 10^−3^ mmol) of component Y in 9 mL of DMAc and 20 mL toluene solvents. Then, 0.028 g (0.2 mmol) of K_2_CO_3_ was gradually added to the mixture while magnetic stirring. Next, the mixture was heated under reflux (300 rpm) at 120–140 °C for 10 h to dehydrate the system. The reaction temperature was then increased to 165 °C, where it was maintained for 14 h. During that time, the solution became very viscous [19]. The solid product was cleaned with a mixture of methanol, acetone, and DI water to remove the low molecular weight substances and dried to furnish a XY block copolymer. XY: ^1^H NMR (600 MHz, DMSO-d_6_) *δ* 7.83–7.69, 7.5–7.44, 7.4–7.3, 7.3–6.9. Afterwards, the obtained XY block copolymer was soaked in 0.5 M H_2_SO_4_ for 12 h to exchange the Na^+^ with H^+^ ions.

### 2.4. Membrane Preparation

**Scheme 1:**
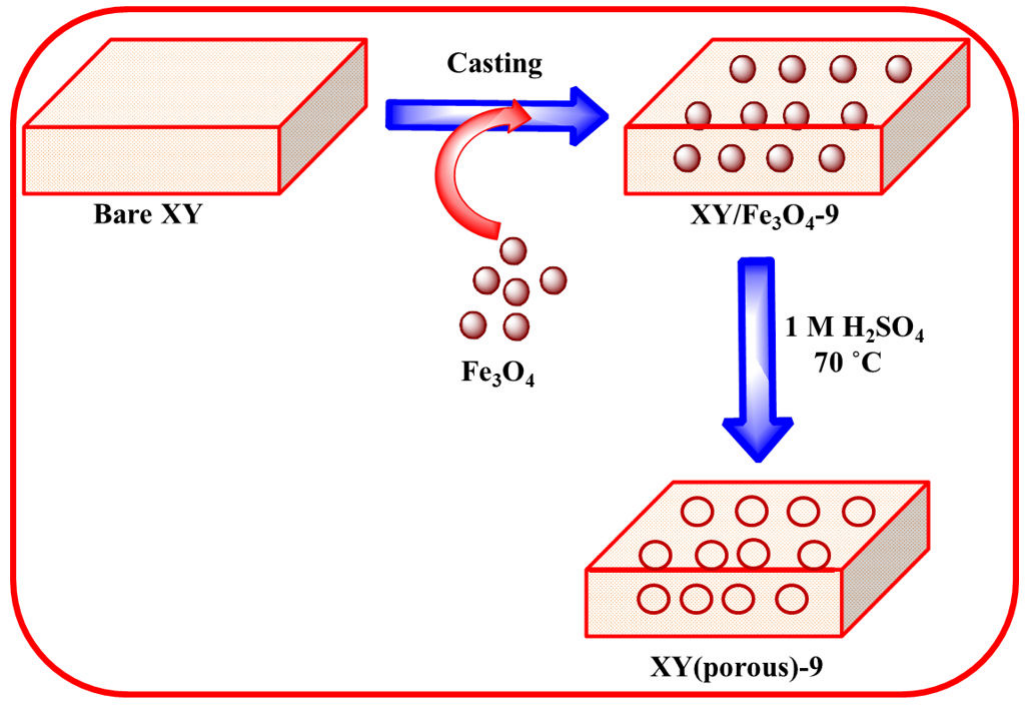
Preparation process of XY(porous)-9 membrane.

**Figure 1:**
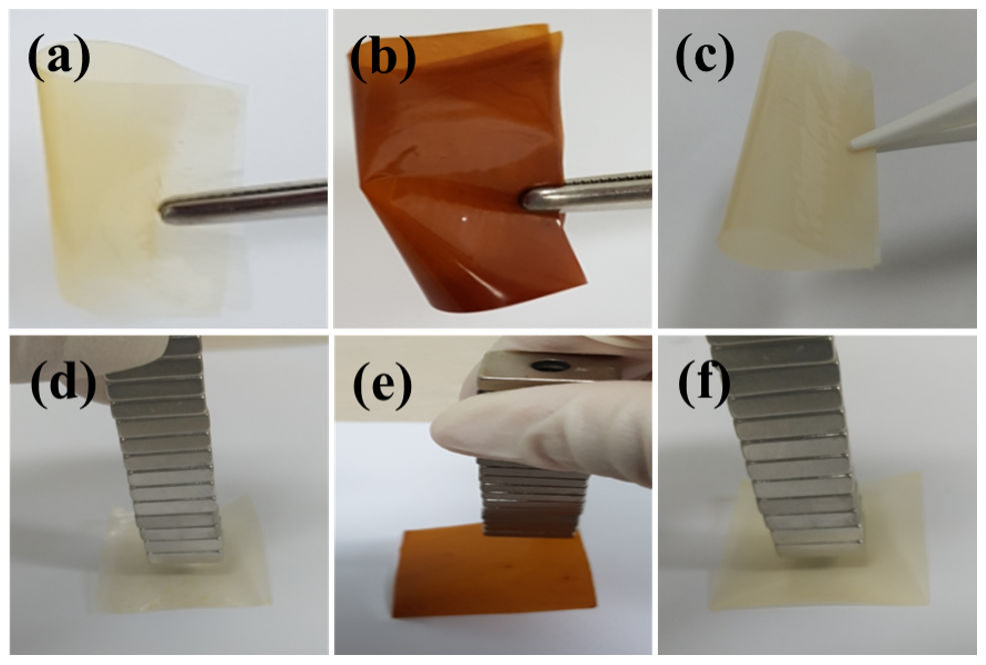
Digital photographic images (**a**,**d**) XY, (**b**,**e**) XY/Fe_3_O_4_-9, and (**c**,**f**) XY(porous)-9 membranes.

Fe_3_O_4_ nanoparticles were prepared using a co-precipitation method as previously described in the literature [20]. Scheme 1 shows the preparation process of composite membrane. Approximately, 0.5 g of the XY block copolymer was initially dissolved in 10 mL DMF using magnetic stirring. Afterward, 3 wt%, 9 wt%, or 12 wt% of Fe_3_O_4_ was dispersed into the solution by sonication to obtain a composite solution. The resulting solution was stirred at 70 °C for desired time to prepare the casting solution. Then, the obtained solution was poured into a glass dish and dried in an oven at 70 °C for 12 h to evaporate the solvent. The prepared composite membrane was peeled off the glass dish via immersion in DI water. Next, the composite membrane was immersed in 1 M H_2_SO_4_ at 70 °C for 1 h to dissolve Fe_3_O_4_ nanoparticles and create pores in the membrane. Finally, the membranes were again immersed into DI water to uptake water in the pores. The XY/Fe_3_O_4_-9 membrane is brown in color and magnetically active (Figure 1b,e), while the bare and XY(porous)-9 membranes are yellow in color and magnetically inactive (Figure 1a,d,c,f). The thickness of the membranes was measured at least three points and was 50–60 µm. 

## 3. Characterizations

The below mentioned instruments except SAXS, GPC and UTM were installed in the center for university-wide research facilities (CURF) at Chonbuk national university (CBNU).

### 3.1. Structural Characterizations

Proton nuclear magnetic resonance (^1^H NMR, JNM-ECA600-600 MHz) was used to ensure the chemical structure of the polymers with anhydrous *DMSO-d_6_* as solvent. Fourier transform infrared spectroscopy (FT-IR, Perkin Elmer, Frontier) was utilized to examine the functional groups in the membranes. High-resolution X-ray diffractometry (XRD, X’Pert MRD with CuKα radiation) was used to analyze the crystalline structure of the membrane samples. Small angle X-ray scattering (SAXS, EMPYREAN, Malvern Panalytical) was used to measure the average ionic cluster dimensions. Gel permeation chromatography (GPC, HLC‒8320GPC‒Tosoh Corporation, Tokyo, Japan) was used to measure the molecular weights of the synthesized oligomers and polymers. 

### 3.2. Morphological Characterizations

The morphological properties of the prepared membranes were studied using a field emission scanning electron microscope (FE-SEM, SUPRA 40VP) furnished with an energy dispersive X-ray (EDX) spectrometer.

### 3.3. Thermal and Mechanical Characterizations

The thermogravimetric analysis (TGA) was performed with a Q50 thermal instrument under a N_2_ atmosphere at a heating rate of 5 °C/min to examine the thermal stability of membrane and filler samples; and a LR5K plus 5 kN universal testing machine (UTM, Ametek Lioyd Instruments, Ltd.) was used to test the mechanical properties of dry membranes (30 mm × 15 mm) at a strain rate of 10 mm/min. We used two different samples for each membrane to check the reproducibility of curves.

## 4. Measurements

### 4.1. Water Uptake

Water uptake measurements were performed to examine the water retention ability of membranes. The dry membrane samples were weighed and immersed into DI water and kept for 24 h at 30 °C. Afterwards, the wet membrane samples were removed from the DI water and weighed again. The water uptake of the membrane calculated using the following formula [21]:

(1) $$\mathrm{Water}\;\mathrm{uptake}\;\mathrm{of}\;\mathrm{the}\;\mathrm{membrane}\;(\%)\;=\;\left[\frac{W_{\mathrm{wet}}-W_{\mathrm{dry}}}{W_{\mathrm{dry}}}\right]\times \;100,$$

where W_wet_ is the weight (mg) of the wet membrane and W_dry_ is the weight (mg) of the dry membrane.

### 4.2. Swelling Ratio

The swelling ratio in length (S_L_), area (S_A_), and volume (S_V_) was measured according to the change in volume of the membrane samples before and after water uptake. The swelling ratio of the membrane samples was calculated with the following formula [22,23,24]:

(2) $$\mathrm{Swelling}\;\mathrm{ratio}\;\mathrm{in}\;\mathrm{length}\;=\;\left[\frac{L_{\mathrm{wet}}-{\;L}_{\mathrm{dry}}}{L_{\mathrm{dry}}}\right]\times \;100,$$

(3) $$\mathrm{Swelling}\;\mathrm{ratio}\;\mathrm{in}\;\mathrm{area}\;=\;\left[\frac{A_{\mathrm{wet}}-{\;A}_{\mathrm{dry}}}{A_{\mathrm{dry}}}\right]\times \;100,$$

(4) $$\mathrm{Swelling}\;\mathrm{ratio}\;\mathrm{in}\;\mathrm{volume}\;=\;\left[\frac{V_{\mathrm{wet}}-{\;V}_{\mathrm{dry}}}{V_{\mathrm{dry}}}\right]\times \;100,$$

where L_wet_, A_wet_, and V_wet_ are the length, area, and volume of the wet membranes; and L_dry_, A_dry_, and V_dry_ are the length, area, and volume of the dry membranes.

### 4.3. Ion Exchange Capacity

Ion exchange capacity (IEC) determines the number of milliequivalents of ions that are present per 1 g of dry membrane [25]. The IECs of the prepared membranes were determined using a titration method. To perform this measurement, the dried membrane was soaked in a 0.1 M NaCl solution for 24 h to exchange H^+^ with Na^+^ ions. Then, the liberated H^+^ ions were titrated with a 0.01 M NaOH solution using phenolphthalein as indicator. The IEC was calculated according to the following equation:

(5) $$\mathrm{IEC}\;\mathrm{of}\;\mathrm{the}\;\mathrm{membrane}\;(\mathrm{meq}/g)\;=\;\left[\frac{\mathrm{Volume}\;\mathrm{of}\;\mathrm{NaOH}\;\mathrm{consumed}\;\times \;\mathrm{Consendration}\;\mathrm{NaOH}}{\mathrm{Weight}\;\mathrm{of}\;\mathrm{dry}\;\mathrm{membrane}}\right].$$

### 4.4. Contact Angle

The contact angle measurements were performed on the prepared samples using a DSA10 instrument (Kruss GmbH, Germany) to identify the wettability or hydrophilicity of membrane surface.

### 4.5. Proton Conductivity 

The proton conductivity of membrane samples was measured at 100% RH and different temperatures ranging from 40 to 80 °C using a four-electrode BekkTech cell equipped with an alternating current impedance spectroscope [26,27,28]. Proton conductivity (σ) was calculated via the following formula:

(6) $$\mathrm{Proton}\;\mathrm{conductivity}\;\mathrm{of}\;\mathrm{the}\;\mathrm{membrane}\;(\mathrm{mS}/\mathrm{cm})\;=\;\left[\frac{L}{\mathrm{RTW}}\right],$$

where L (cm) is the distance between the electrodes, R (ohm) is the resistance of membrane, T (cm) is the thickness of the membrane, and W (cm) is the width of the membrane.

### 4.6. Fabrication of Membrane Electrode Assembly and PEFC Test 

The PEFC performance was tested using a fuel cell machine (Horizon Fuel Cell Technologies, Model: SKFC-TS001, South Korea) at 60 °C and 60% relative humidity under atmospheric pressure. In brief, the membrane–electrode assembly (MEA) was prepared through the conventional hot-pressing technique with commercial gas diffusion electrodes (GDE) (Pt catalyst loading of 0.3 mg/cm^2^). Membranes were tightened with the GDEs and hot-pressed at 100 °C for 3 min with a pressure of 100 kg/cm^2^. The active area of the obtained MEA was 5 cm^2^, which was fixed in PEFC device and tested the performance. H_2_ gas and air were passed at 100 and 400 mL/min for the corresponding anode and cathode, respectively.

## 5. Results and Discussion

### 5.1. Structural Properties

**Figure 2:**
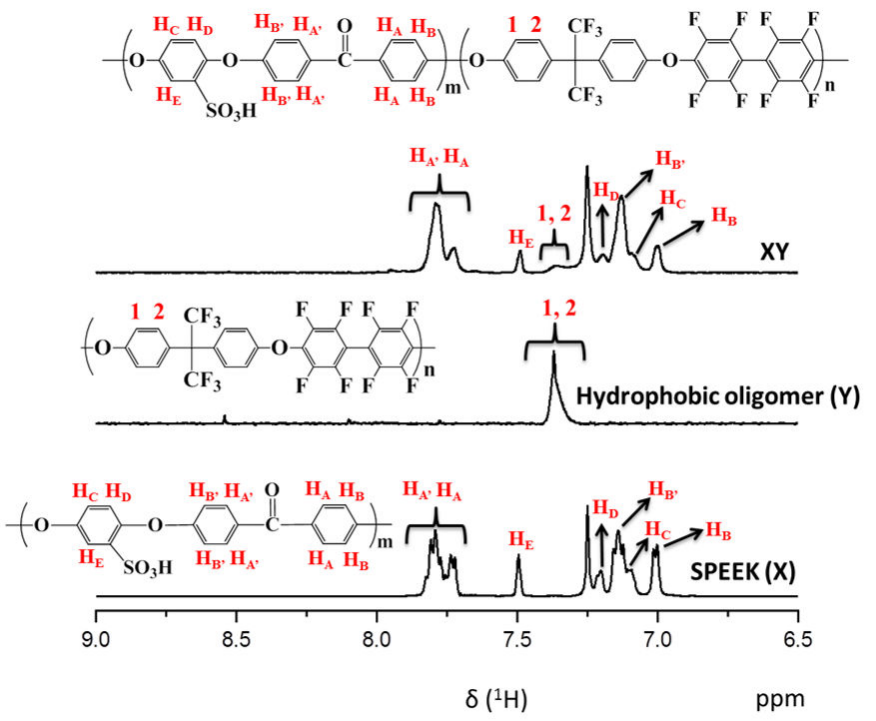
Proton nuclear magnetic resonance (^1^H NMR) spectra of X, Y, and XY block copolymers. SPEEK—sulfonated poly(ether ether ketone).

**Figure 3:**
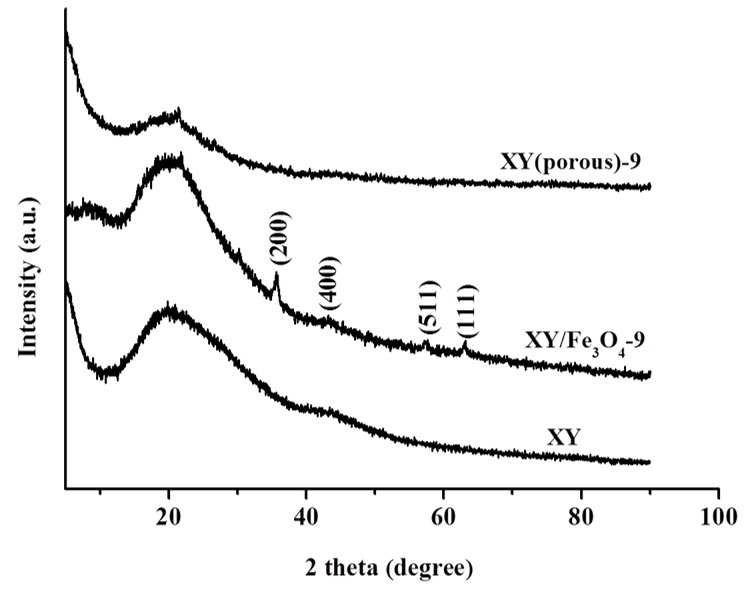
X-ray diffractometry (XRD) patterns of bare XY, XY/Fe_3_O_4_-9, and XY(porous)-9 membranes.

**Figure 4:**
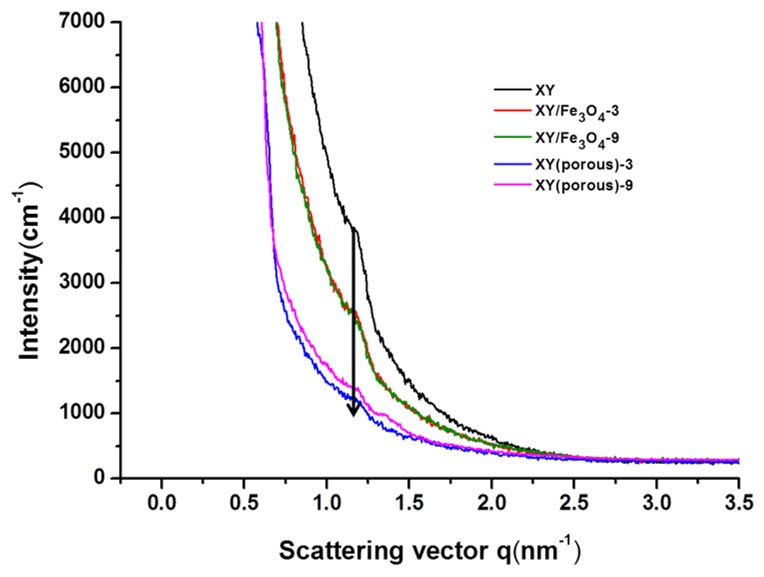
Small angle X-ray scattering (SAXS) patterns of the bare XY, composite, and porous membranes.

We controlled the X and Y components in the prepared XY block copolymers at a 1:1 molar ratio. X, Y, and XY block copolymers exhibited molecular weights (M_w_) of 142,800, 20,100, and 164,100, respectively, and the polydispersity index (PDI) (*M*_w_/*M*_n_) values for the X, Y, and XY block copolymers were 2.2, 4.1, and 3.3, respectively (Table 1). The chemical structures of synthesized polymers identified by ^1^H NMR spectroscopy are shown in Figure 2. The proton peaks of X were observed between 6.9 to 7.9 ppm in the ^1^H NMR spectrum, corresponding to aromatic protons. In addition, the successful attachment of SO_3_H groups was verified by the peak at 7.5 ppm, and the degree of sulfonation (DS) was calculated as 42% using this peak. Similarly, the XY block copolymer had significant signals in the range of 6.9 to 7.9 ppm, which arose from the aromatic protons of both SPEEK and the hydrophobic units. Functional groups of pristine and composite membrane were identified by FT-IR (Figure S2). The bands found about 1004 and 1015 cm^−1^ in the XY spectrum were consigned to the symmetric stretching vibration of in-chain diphenyl ether (Ar–O–Ar) existing in the para-substituted benzene ring of the Y part [29]. The peaks at 1223, 1069, and 1009 cm^−1^ are associated with the asymmetric and symmetric stretching of sulfone in SO_3_H from the X part. These bands confirmed the formation of the XY structure. Along with the aforementioned bands, the Fe–O stretching at 520 cm^−1^ was also observed in the composite membrane [30], indicating the formation of the composite membrane. Again, the Fe–O stretching disappeared in the case of the XY/(porous)-9 membrane, verifying the successful removal of Fe_3_O_4_ by acid treatment. The XRD patterns of XY, XY/Fe_3_O_4_-9, and XY(porous)-9 were investigated to evaluate the crystalline structure of prepared materials (Figure 3). When compared with the XY membrane, the XY/Fe_3_O_4_-9 membrane exhibits additional peaks of 200, 400, 511, and 111 planes related to Fe_3_O_4_. Such a result confirms the successful formation of the composite structure. As further validation, the aforementioned Fe_3_O_4_ planes disappeared for the XY(porous)-9 sample as a result of the acid treatment. On the other hand, the ionic cluster dimensions of bare and composite membranes were confirmed using SAXS [31], and similar ionic cluster dimensions were observed in both the bare XY and composite membranes, as shown in Figure 4.

### 5.2. Morphological Properties

**Figure 5:**
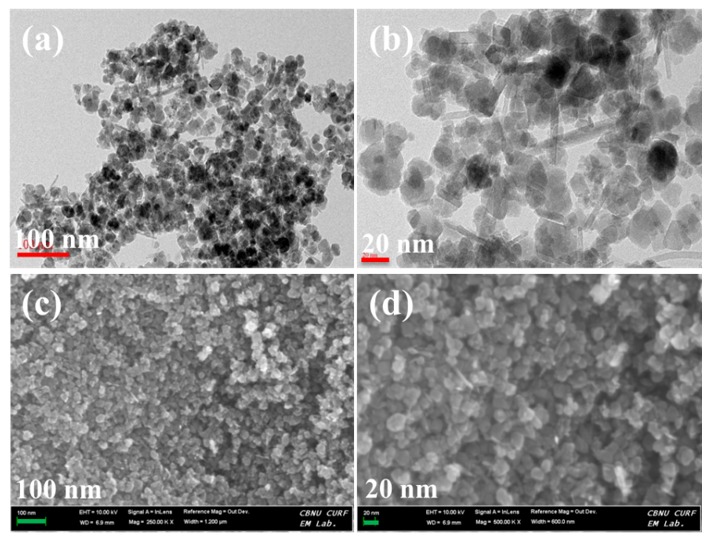
(**a**,**b**) TEM images and (**c**,**d**) field emission scanning electron microscope (FE-SEM) images of Fe_3_O_4_ nanoparticles.

**Figure 6:**
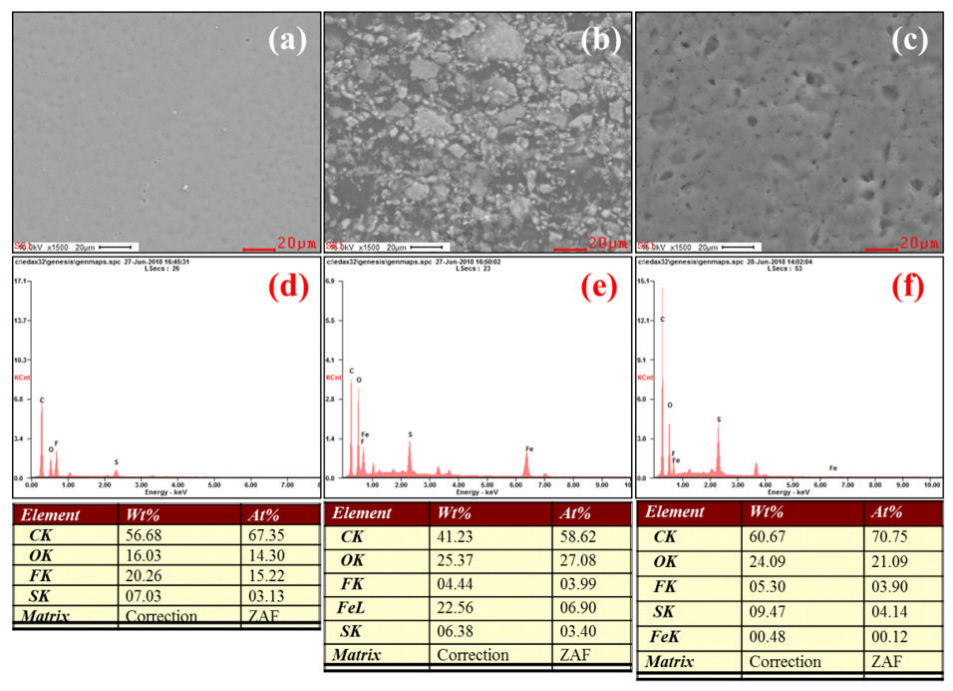
FE-SEM images and energy dispersive X-ray (EDX) spectra of membranes of (**a**,**d**) XY, (**b**,**e**) XY/Fe_3_O_4_-9, and (**c**,**f**) XY(porous)-9.

**Figure 7:**
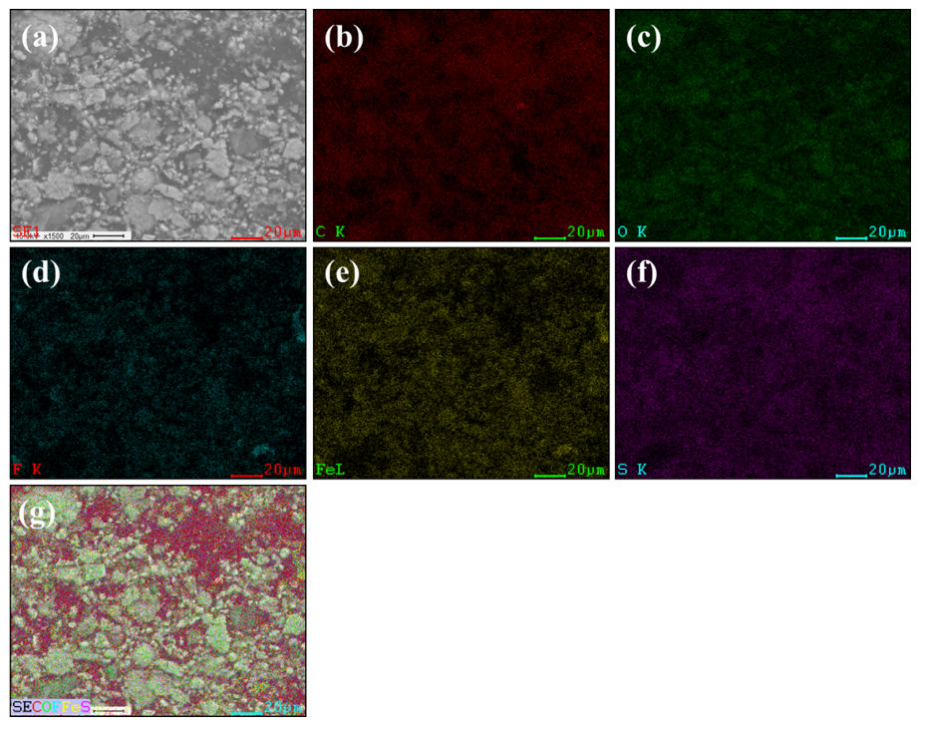
(**a**) FE-SEM selected area of XY/Fe_3_O_4_-9, (**b**) carbon, (**c**) oxygen, (**d**) fluorine, (**e**) iron, (**f**) sulfur, and (**g**) overlapping of elements.

The morphologies of Fe_3_O_4_ nanoparticles and membranes were investigated using TEM and FE-SEM, and the results are demonstrated in Figure 5 and Figure 6. Using this analysis, we observed the Fe_3_O_4_ nanoparticles with the size of 30 nm, demonstrating that the successful formation of Fe_3_O_4_ nanoparticles via the co-precipitation method. The XY membrane shows a clean morphology where there are no cracks, pinholes, or perforations observed, indicating the formation of an efficient membrane (Figure 6a). Compared with the bare XY membrane, the composite membrane has a rough surface due to the existence of Fe_3_O_4_ nanoparticles (Figure 6b). After treatment with 1 M H_2_SO_4_ at 70 °C, the Fe_3_O_4_ nanoparticles dissolved and, consequently, pores were generated on the surface (Figure 6c). Such pores act as nano-channels for the efficient transfer of protons. To investigate the dispersibility of Fe_3_O_4_ or pores across the cross-section of the membranes, the cross-sectional FE-SEM images were observed, as given in Figure S1a–i (Supplementary Materials). The Fe_3_O_4_ nanoparticles and pores were randomly dispersed in the cross-section of the membranes. EDX elemental analysis was performed to investigate the porous structure in the membranes. As shown in Figure 6f, the Fe_3_O_4_ nanoparticles were dissolved successfully using an acid treatment. According to the elemental mapping images (Figure 7), the Fe_3_O_4_ particles were spread across the surface of membrane, which might increase the membrane porosity. 

### 5.3. Thermal and Mechanical Properties

**Figure 8:**
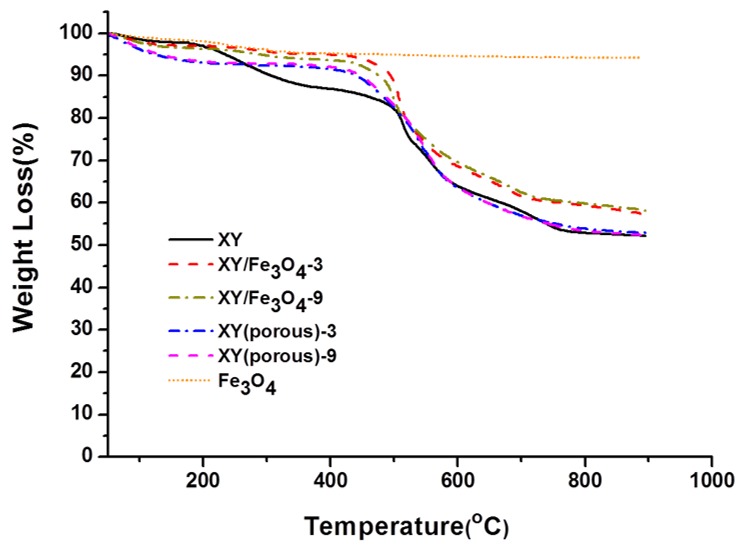
Thermogravimetric analysis (TGA) curves of Fe_3_O_4_ nanoparticles, and bare XY, composite, and porous membranes.

**Figure 9:**
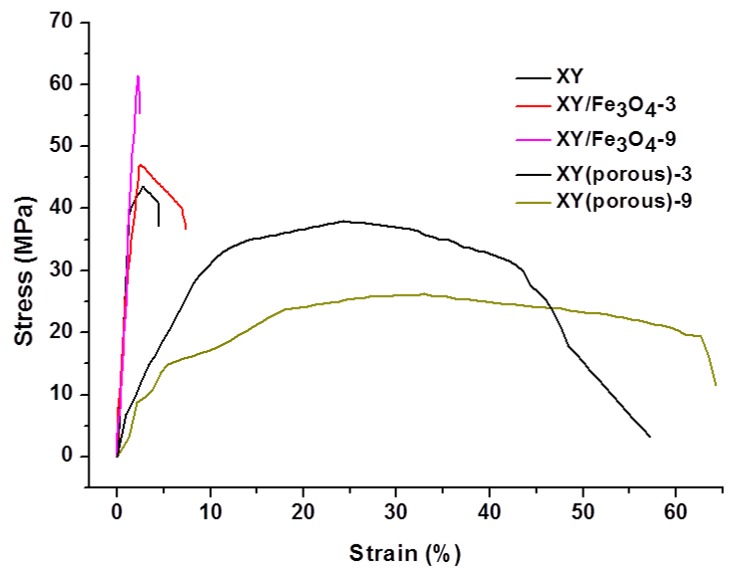
Stress–strain curves of the bare XY, composite, and porous membranes.

The TGA results are shown in Figure 8. The TGA curves show three stages of thermal degradation in the prepared samples. The first stage of degradation occurred in the temperatures range of 30–200 °C due to the thermal decomposition of free water molecules in samples [32]. The second stage of degradation took place between 220–400 °C and can be attributed to the thermal decomposition of SO_3_H groups in the samples [33,34]. The final degradation stage occurred at temperatures above 500 °C due to the decomposition of the polymer backbone. The prepared XY/Fe_3_O_4_-9 composite membrane showed higher thermal stability than the bare XY membrane because of the presence of thermally-stable Fe_3_O_4_ nano-fillers. After removal of Fe_3_O_4_ nanoparticles via acid treatment, the porous XY membrane degraded rapidly. The mechanical stabilities of membranes were measured at a dry state at room temperature (Figure 9). The stress of XY is acceptable; however, once the XY backbone was blended with Fe_3_O_4_, the increased stress was observed, which may be ascribed to electrostatic interactions between the polymer chains and Fe_3_O_4_. Nevertheless, a lower strain in the XY/Fe_3_O_4_-9 indicates its high brittleness. On other hand, the XY(porous)-9 membrane exhibits high strain because of its high flexibility across the membrane provided by the porous architecture [18]. Thus, it is believed that XY(porous)-9 has a high plasticization nature, which causes the membrane to have high strain.

### 5.4. Water Uptake, Swelling Ratio, IEC, and Contact Angle

**Figure 10:**
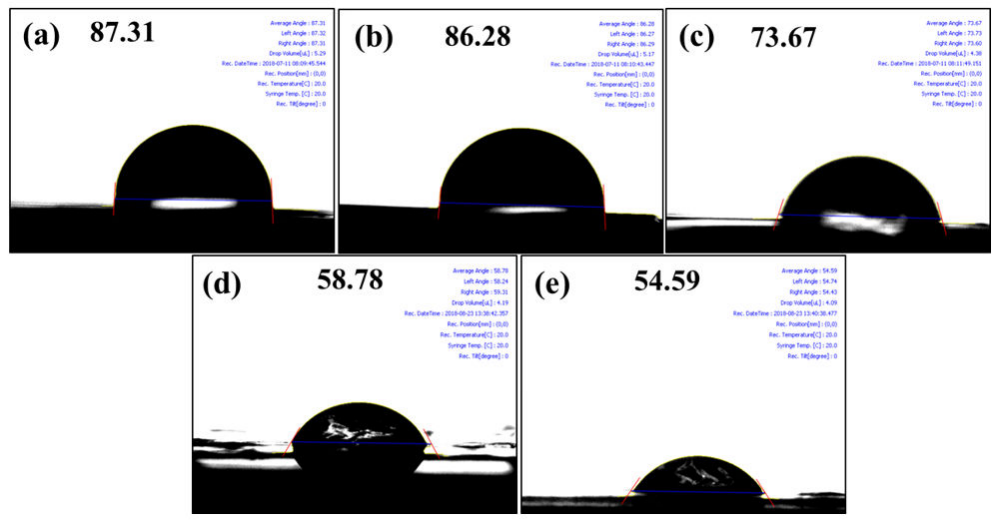
Water contact angle images of (**a**) bare XY, (**b**) XY/Fe_3_O_4_-3, (**c**) XY/Fe_3_O_4_-9, (**d**) XY(porous)-3, and (**e**) XY(porous)-9 membranes.

The water uptake, swelling ratio, IEC, and contact angle measurements are essential factors in determining the suitability of a membrane for PEFC applications (Table 2) [22]. The proton conductivity of membranes is closely correlated to water uptake; a membrane that contains a greater quantity of water molecules can facilitate proton transport via vehicle mechanism. The bare XY membrane shows a water uptake of 11.9%, while water uptakes of 16.7% and 26.6% were observed for XY/Fe_3_O_4_-9 and XY(porous)-9 membranes, respectively. The higher water uptake for the porous membrane is the result of the existence of pores in membranes, which accommodate the water molecules. In general, the amount of water absorption depends directly on the amount of hydrophilic channels in a membrane, and the amount of hydrophilic channels in a membrane is directly proportional to the degree of sulfonation. Likewise, the swelling ratio exhibits a similar dependency to that of water uptake. Compared with the bare XY membrane, the XY/Fe_3_O_4_-9 and XY(porous)-9 membranes revealed high swelling degrees in length, area, and volume, owing to the higher water uptake. Furthermore, IEC is closely related to the density of sulfonic acid groups in the membrane. The IEC value of the XY membrane was 1.24 meq/g, and it was 1.41 meq/g and 1.81 meq/g for the XY/Fe_3_O_4_-9 and XY(porous)-9 membranes, respectively. Hence, the proton conductivity of the XY(porous)-9 membrane (124.2 mS/cm) is much higher than that of the bare XY membrane (71.5 mS/cm) and XY/Fe_3_O_4_-9 (83.9 mS/cm) at 80 °C. Contact angle images of prepared membranes are shown in Figure 10. When compared with the XY membrane, the XY/Fe_3_O_4_-9 membrane sample shows a lower contact angle due to the existence of Fe_3_O_4_ nanoparticles. Interestingly, the XY(porous)-9 membrane exhibits an even lower contact angle, likely because of the pores in the membrane

### 5.5. Proton Conductivity and PEFC Performance and Durability

**Figure 11:**
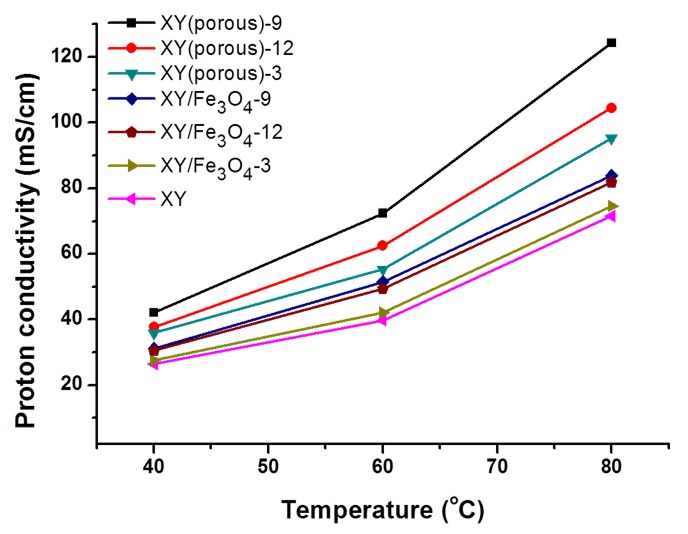
Proton conductivity plots of the bare XY, composite, and porous membranes.

**Figure 12:**
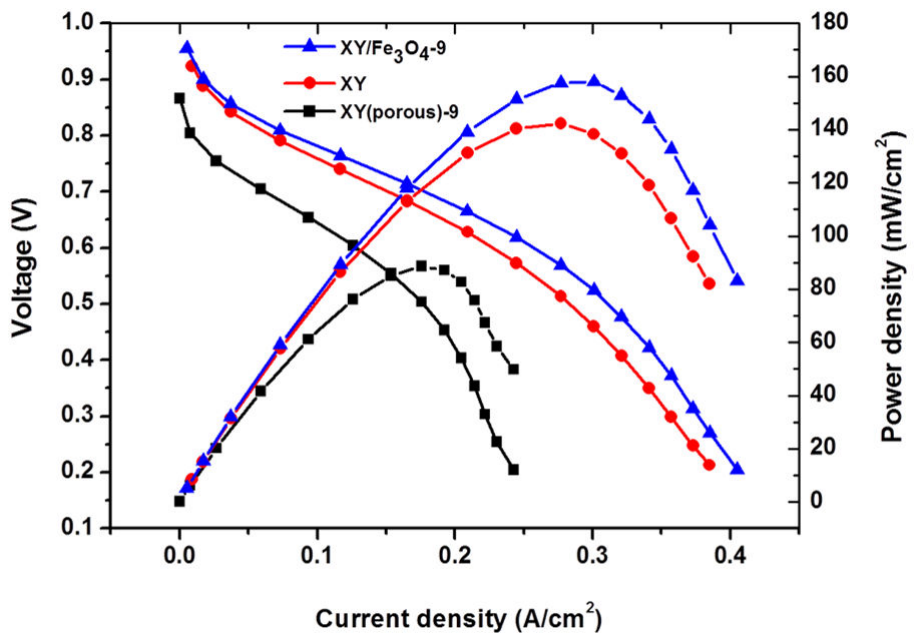
Polymer electrolyte fuel cell (PEFC) performance of the bare XY, composite, and porous membranes.

In PEMFC, the proton conductivity of a membrane is considered as one of the most influential properties. It is believed that the enhancement in proton conductivity of a membrane leads to improvements in the total power density of a cell [35]. Typically, PEM proton conductivity follows two mechanisms: (i) the vehicle mechanism and (ii) the Grotthuss mechanism [36]. In the vehicle mechanism, hydrogen bonding in the polymer matrix is considered to be responsible for the transportation of H^+^ ions [37]. In high humidity conditions, the water molecules absorbed onto the wet membrane will react with the hydrogen bonds in polymer matrix and become hydronium (H_3_O^+^) ions. In the Grotthuss mechanism, the H_3_O^+^ ions are thought to be responsible for the transportation of protons in membrane [38]. In this study, all of the prepared membranes exhibited both types of proton transport mechanisms. Figure 11 shows the temperature dependency of proton conductivity in the prepared membranes. The proton conductivity was observed to increase with increasing cell temperatures for all prepared membranes. From the curves, it is apparent that the XY(porous)-9 has a higher proton conductivity (124.2 mS/cm at 80 °C) than the other prepared membranes as a result of the domination of the vehicle mechanism. Furthermore, the nano-pores in polymer matrix may facilitate the absorption of excess water molecules and, consequently, facilitate proton transport via the vehicle mechanism. In the case of the XY(porous)-12 membrane, however, the proton conductivity is lower because of the aggregated pore structure. Therefore, we hypothesize that the high distribution and aggregation of pores may lower the proton conductivity of the membrane. On the other side, XY/Fe_3_O_4_ membranes exhibit higher conductivity than the bare XY membrane, indicating that Fe_3_O_4_ nanoparticles help to facilitate the conductivity in the membrane. The PEFC performance of bare XY, XY/Fe_3_O_4_-9, and XY(porous)-9 membranes was compared at 60 °C under 60% relative humidity, and the respective polarization curves are plotted in Figure 12. XY/Fe_3_O_4_-9 achieved the highest maximum power density of 159 mW/cm^2^ at a load current density of 299 mA/cm^2^, followed by bare XY (142 mW/cm^2^) and XY(porous)-9 (88.5 mW/cm^2^) at the load current densities of 276 mA/cm^2^ and 176 mA/cm^2^, respectively. The primary reason for the lower power density of the porous membrane might be the high fuel crossover through the membrane. The pores in the XY(porous)-9 membrane allow the protons along with hydrogen, hence the power density the membrane is observed to be lower. The durability data of the prepared membranes were recorded at 60 °C under 60% relative humidity over 70 h, and the obtained results are provided in Figure S3. The total voltage decays of pristine XY, XY(Fe_3_O_4_)-9, and XY/(porous)-9 membranes are 0.35, 0.76, and 0.27 V, respectively. Compared with pristine XY and XY/(porous)-9 membranes, the composite membrane exhibits a high voltage decay because of the existence of Fe_3_O_4_. Fe^3+^ and Fe^4+^ ions in the Fe_3_O_4_ catalyze the radicals generation in the cell. Such radicals attach the membranes and accelerate the chemical degradation. Consequently, the membranes exhibit high voltage decay.

## 6. Conclusions

In this study, hygroscopic Fe_3_O_4_ nanoparticles were synthesized via the co-precipitation method and doped onto an XY block copolymer matrix to fabricate a novel PEM. Furthermore, we created a porous structure in the XY matrix by removing Fe_3_O_4_ nanoparticles by immersing a membrane in a 1 M H_2_SO_4_ solution at 70 °C for 1 h. When comparing the bare XY and XY(porous)-9 membranes, the XY/Fe_3_O_4_-9 membrane shows high stress and a significant improvement in mechanical strength and thermal stability. Although XY(porous)-9 membrane show improved water uptake, proton conductivity, and IEC, the PEFC performance of XY(porous)-9 is observed to be lower. Thus, we justify that the XY/Fe_3_O_4_-9 membrane is more suitable for PEFC device applications than the XY(porous)-9 membrane. As a result, the present study provides valuable information about the fabrication and comparison of physiochemical, thermomechanical, and electrochemical properties of the XY/Fe_3_O_4_-9 and XY(porous)-9 membranes. Thus, this study might assist to understand the importance of the XY/Fe_3_O_4_-9 or XY(porous)-9 membrane for their wide application in PEFCs.

## Figures and Tables

**Table 1:** GPC analysis of prepared polymers.

Polymer	M_n_	M_w_	M_z_	M_w_/M_n_ (PDI)
X	62,600	142,800	282,000	2.2
Y	4800	20,100	47,800	4.1
XY	48,900	164,100	455,400	3.3

**Table 2:** Physiochemical properties of prepared membranes. IEC—ion exchange capacity.

Membrane	Water Uptake (%)	Swelling Ratio (%)			IEC (meq g^−1^)
	Mass	Length (S_L_*)*	Area (S_A_)	Volume (S_V_)	
XY	11.9	4.6	9.7	12.9	1.24
XY/Fe_3_O_4_-3	14.2	5.0	10.2	13.9	1.33
XY/Fe_3_O_4_-9	16.7	6.7	13.7	16.4	1.41
XY/porous-3	22.3	7.3	15.0	18.7	1.60
XY/porous-9	26.6	7.5	16.0	20.9	1.81
Nafion-117	13.8	9.2	18.9	24.7	0.94

## Supplementary Materials

The supplementary materials are available online at https://www.mdpi.com/2073-4360/11/3/536/s1.
