# Supplementary material for: A Comparative Study on Physiochemical, Thermomechanical, and Electrochemical Properties of Sulfonated Poly(Ether Ether Ketone) Block Copolymer Membranes with and without Fe3O4 Nanoparticles
Source: Polymers (Basel). 2019 Mar 21;11(3):536. doi: 10.3390/polym11030536 (PMC6473931; doi:10.3390/polym11030536)
Supplement: Supplementary file 1 [file polymers-11-00536-s001.pdf]

A comparative study on physiochemical, thermomechanical and electrochemical properties of sulfonated poly(ether ether ketone) block copolymer membranes with and without Fe_3O_4 nanoparticles

Supporting information

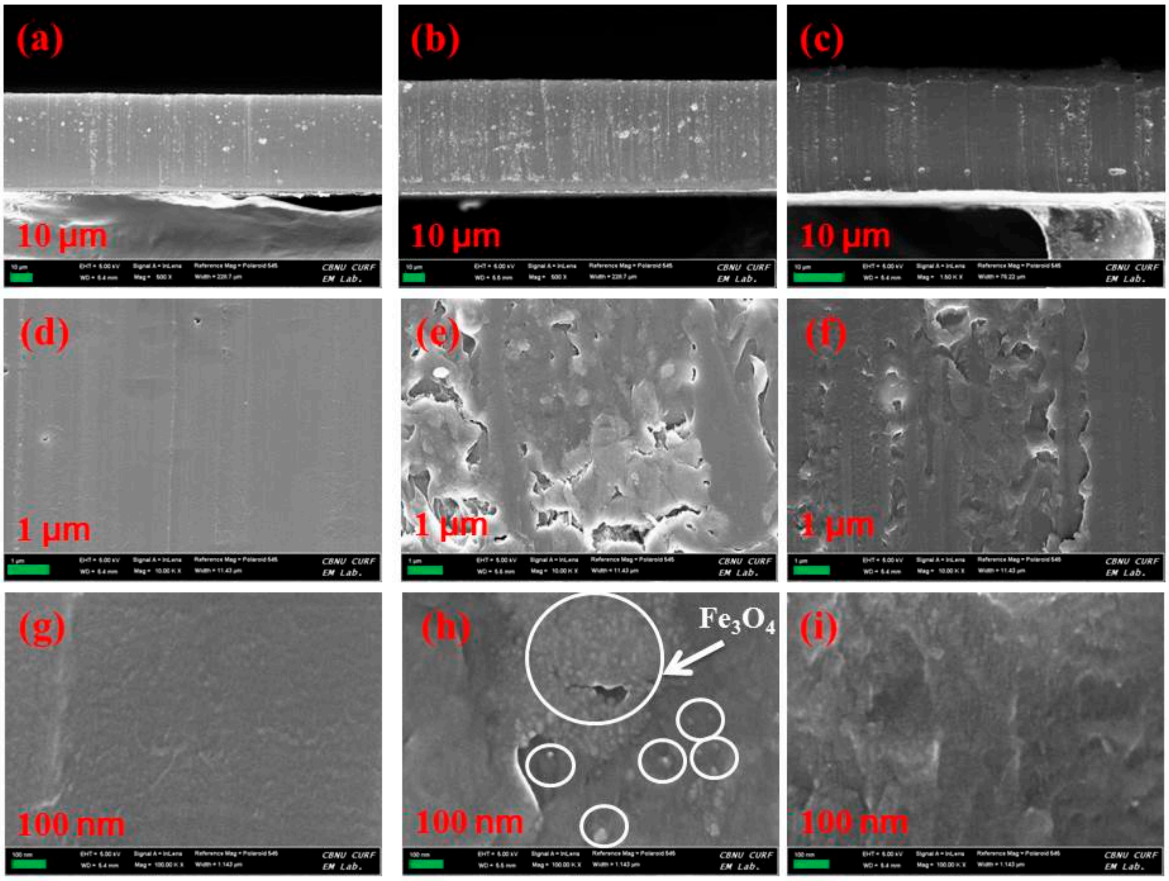

Fig. S1. Cross section FE-SEM images of (a, d and g) XY, (b, e and h) XY/Fe_3O_4-9 and (c, f and h) XY/(porous)-9 membranes.

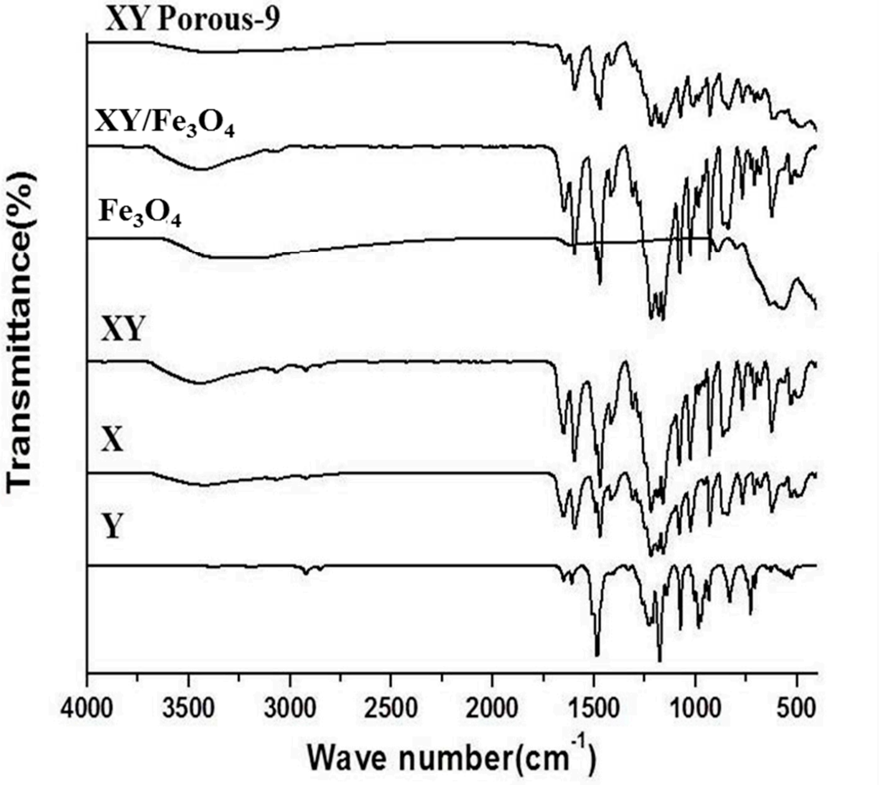

Fig. S2. FT-IR spectra of X, Y, composite and porous membranes.

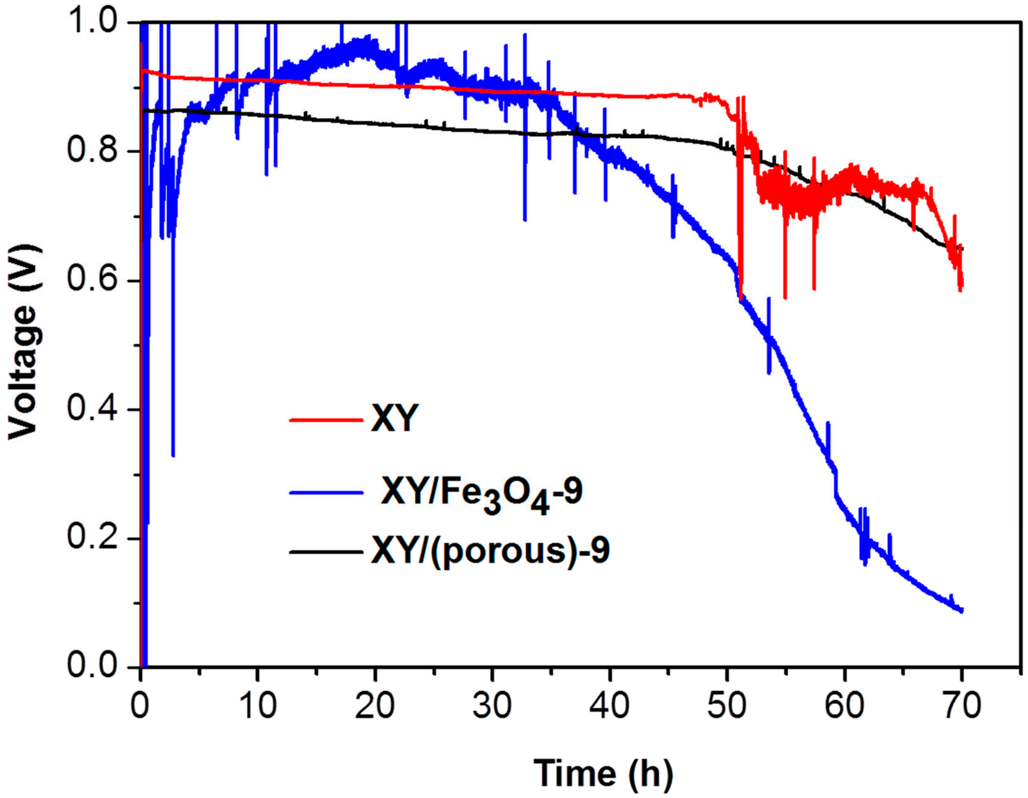

Fig. S3. Durability curves of XY, XY /Fe_3O_4-9 and XY/(porous)-9 membranes.
